# Feedback of mechanical effectiveness induces adaptations in motor modules during cycling

**DOI:** 10.3389/fncom.2013.00035

**Published:** 2013-04-17

**Authors:** Cristiano De Marchis, Maurizio Schmid, Daniele Bibbo, Anna Margherita Castronovo, Tommaso D'Alessio, Silvia Conforto

**Affiliations:** Department of Engineering, Roma TRE UniversityRome, Italy

**Keywords:** muscle synergies, modularity, cycling, biomechanical function, instrumented pedals, pedaling effectiveness, biofeedback

## Abstract

Recent studies have reported evidence that the motor system may rely on a modular organization, even if this behavior has yet to be confirmed during motor adaptation. The aim of the present study is to investigate the modular motor control mechanisms underlying the execution of pedaling by untrained subjects in different biomechanical conditions. We use the muscle synergies framework to characterize the muscle coordination of 11 subjects pedaling under two different conditions. The first one consists of a pedaling exercise with a strategy freely chosen by the subjects (Preferred Pedaling Technique, PPT), while the second condition constrains the gesture by means of a real time visual feedback of mechanical effectiveness (Effective Pedaling Technique, EPT). Pedal forces, recorded using a pair of instrumented pedals, were used to calculate the Index of Effectiveness (*IE*). EMG signals were recorded from eight muscles of the dominant leg and Non-negative Matrix Factorization (NMF) was applied for the extraction of muscle synergies. All the synergy vectors, extracted cycle by cycle for each subject, were pooled across subjects and conditions and underwent a 2-dimensional Sammon's non-linear mapping. Seven representative clusters were identified on the Sammon's projection, and the corresponding eight-dimensional synergy vectors were used to reconstruct the repertoire of muscle activation for all subjects and all pedaling conditions (*VAF* > 0.8 for each individual muscle pattern). Only 5 out of the 7 identified modules were used by the subjects during the PPT pedaling condition, while 2 additional modules were found specific for the pedaling condition EPT. The temporal recruitment of three identified modules was highly correlated with *IE*. The structure of the identified modules was found similar to that extracted in other studies of human walking, partly confirming the existence of shared and task specific muscle synergies, and providing further evidence on the modularity of the motor system.

## Introduction

The study of the neuro-physiological mechanisms underlying movement production has a long fascinating history (Bernstein, 1967). In the last decade the scientific community has been focusing its attention on the possibility of simplifying the role of the central nervous system (CNS) for the production of movement, by hypothesizing that the complex muscle coordination shown during the execution of a variety of motor acts relies on a simple combination of motor modules (d'Avella et al., 2003). Experimental evidence has been provided that surface ElectroMyoGraphic signals (sEMG) recorded from many muscles during the execution of movement can be represented by the combination of a reduced number of muscle synergies. The latter constitute modules of muscle co-activation that—flexibly combined through amplitude scaling and time shifting mechanisms—can accurately reconstruct the repertoire of muscle activation for many motor tasks (d'Avella et al., 2003). Muscle synergies have been investigated in motor tasks like running (Cappellini et al., 2006), postural responses (Torres-Oviedo and Ting, 2007), pedaling (Hug et al., 2010), walking in normal and pathologic conditions (Ivanenko et al., 2004; Clark et al., 2010; Monaco et al., 2010) and upper limb reaching (d'Avella et al., 2008; Cheung et al., 2012). From this background it emerges that the muscle synergies paradigm seems to fairly represent the neural strategies underlying the control of movement, with motor modules characteristic for each task and robustly shared among different subjects, in terms of both temporal and spatial organization of the muscle activity. Moreover, the existence of a few shared and task-specific muscle synergies during the execution of different movements in the freely moving frogs (d'Avella and Bizzi, 2005) and the existence of separate modules during the coordination of locomotion with voluntary actions (Ivanenko et al., 2005), provides further evidence of modularity.

Nevertheless, the fact that muscle synergies actually reflect neural strategies has been criticized, and it has been hypothesized that they simply reflect the biomechanical constraints during movement execution (Kutch and Valero-Cuevas, 2012). As a matter of fact, a neuro-physiological mechanism able to fully justify the muscle synergy model is still lacking (Tresch and Jarc, 2009).

The linkage between muscle coordination and the mechanical outcome of movement has recently provided further insight into the modular control of movement through the use of simulation studies of human gait (Neptune et al., 2009). It has also been shown that scaling in amplitude and shifting in time the same small number of fixed motor modules leads to the satisfaction of altered mechanical task demands, and that the main modifications occur in the recruitment of those modules recognized as responsible of that particular biomechanical sub-function (Cheung et al., 2009; McGowan et al., 2010).

Modification of the mechanical constraint has also been investigated in cycling tasks executed by trained subjects, where it has been shown that the same few modules are shared among subjects and among different pedaling conditions, with limited adaptations in the synergy activation coefficients (Hug et al., 2011).

Even if cycling gesture is a quasi-constrained exercise with controllable experimental conditions, little is known about the effect of the pedaling technique (i.e., a mechanical factor in terms of force orientation on the pedal along the pedaling cycle) on the structure of the muscular coordination and on the underlying structure of the motor modules. Analysis of forces through the use of instrumented pedals can provide an insight about the effect of different pedaling techniques on muscle coordination (Mornieux et al., 2008). Many variables involved in cycling, such as physiological and metabolic factors, could influence the mechanical outcome and they are functionally connected to the evaluation of the athletes' performance (Zameziati et al., 2006). The concept of mechanical effectiveness in cycling is one of these, and it is directly related to the ability of the subject to orient the pedal forces so that all the expressed forces participate to the propulsive action. Index of mechanical effectiveness, defined as the ratio between the tangential force component and the total one, has been used as an indicator of cycling behavior and it has been put in relation with other parameters such as muscular efficiency or metabolic consumption (Mornieux et al., 2006; Zameziati et al., 2006).

In this study we enrolled untrained subjects to investigate whether a change in the pedaling technique, induced by a visual feedback of mechanical effectiveness, is accomplished by neuromuscular adaptations in modular motor control. In order to do this, we described the pedaling gesture from a biomechanical point of view by combining pedal forces, measured by instrumented pedals, and multi-muscle surface EMG recordings. Our main hypothesis is that when passing from a subject freely chosen pedaling technique to a novel one imposed by the visual feedback of mechanical effectiveness, EMG patterns could change altering the synergy recruitment rather than the structure or the number of the motor modules as a consequence of motor adaptation.

## Materials and methods

### Participants

Eleven male voluntary subjects (aged 27.4 ± 2.5 years) participated to the study. Each subject had no previous experience with professional cycling and reported less than 50 km riding in the previous 3 years. None of them reported previous history of lower limb pathology or surgery. The subjects were informed about the possible discomforts deriving from the experimental protocol and agreed to participate through an informed consent. The study was carried out according to the principles of the declaration of Helsinki.

### Pedal forces recordings and feedback of mechanical effectiveness

**Figure 1:**
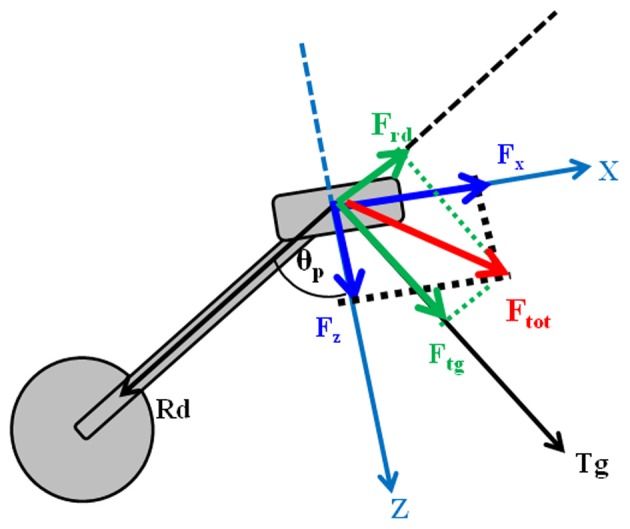
**Pedal forces in the pedal reference system (X-Z) and in the rotating one (Tg-Rd)**.

Pedal forces were recorded by means of a pair of custom two-components instrumented clipless pedals, measuring the two orthogonal components of force *F*_*x*_ and *F*_*z*_ (respectively parallel and orthogonal to the pedal surface as in Figure 1, with an accuracy of 0.1% and a range of 2000 N), together with the angle θ_*p*_ between the direction of the crank arm and a direction orthogonal to the pedal surface (Bibbo et al., 2009a). The pedals use a KEO clip-less fastening. *F*_*x*_ and *F*_*z*_ components of force were acquired using a previously developed recording wireless system (iPED) (Bibbo et al., 2009b) that provides the tangential and radial force components, according to the Equations 2 and 3:

(1) $$F_{\text{tot}}=\sqrt{F_{x}^{2}+F_{z}^{2}}$$

(2) $$F_{\text{tg}}=F_{x}\cos\left(\theta_{p}\right)+F_{z}\sin\left(\theta_{p}\right)$$

(3) $$F_{\text{rd}}=-F_{x}\sin\left(\theta_{p}\right)+F_{z}\cos\left(\theta_{p}\right)$$

The iPED system also provides the index of mechanical effectiveness (*IE*), determined as reported in Equation 4:

(4) $$IE=\frac{\int_{0}^{2\pi }F_{\text{tg}}\left(\theta_{p}\right)d\theta_{p}}{\int_{0}^{2\pi }F_{\text{tot}}\left(\theta_{p}\right)d\theta_{p}}$$

**Figure 2:**
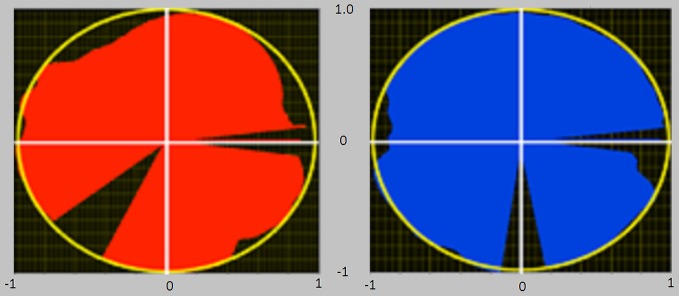
**Visual feedback of the instantaneous mechanical effectiveness, based on a polar plot diagram.** At the end of each completed cycle an image was presented to the subjects, consisting of the polar plots reported in figure. The filling of each circle in different angular sectors/angular positions is proportional to *IE* in that sector/angular position. If the circle is entirely filled (red for left pedal and blue for right pedal), then *IE* equals 1. The polar representation gives information regarding the different phases of the pedaling cycle in which the pedaling technique must be improved.

*IE* is an index theoretically varying in the range [−1, 1] and approaches 1 as the tangential force profile overlaps the total one along the whole pedaling cycle. *IE* was used as a global indicator of performance, while the subjects were provided with a real time visual feedback of instantaneous mechanical effectiveness *IE*_*i*_ drawn on a polar plot (Figure 2), and defined as follows:

(5) $$IE_{i}(\theta_{p})=\frac{F_{\text{tg}}(\theta_{p})}{F_{\text{tot}}(\theta_{p})}$$

*IE*_*i*_ is drawn as a vector whose magnitude is proportional to the instantaneous mechanical effectiveness and whose phase is proportional to the pedal angle.

In this way, the subjects were helped to effectively orient the forces along the pedaling cycle, receiving real time information about which sector of the cycle they had to improve to reach an optimal pedaling technique (Bibbo et al., 2012). An entirely filled circle thus corresponds to *IE* = 1.

### Experimental protocol

The experimental protocol was carried out on an aerodynamically braked cycle-simulator equipped with the instrumented pedals described in the previous section and standard 170 mm cranks. Before the execution of the exercises, the subjects performed a 10-min warming up session. At the end of the warm up the subjects performed a 10 s all-out trial, with the aim of determining the maximum reachable power output (resulting in 634.4 ± 85.5 W). The experimental procedure started after a 3-min rest period and consisted of two different sub-maximal pedaling exercises, each one lasting 2 min: the first exercise consisted in a 2-min pedaling task with a strategy freely chosen by the subject (Preferred Pedaling Technique, PPT). At the end of the first exercise, the subjects, having no previous knowledge about the concept of mechanical effectiveness in cycling, were instructed by the experimenter on how to follow the visual feedback of *IE*_*i*_ and on how to optimally orient the forces on the pedal. After a familiarization with the feedback system, the subjects executed a second pedaling exercise consisting of a 2-min pedaling task aided by feedback (Effective Pedaling Technique, EPT). For both exercises the subjects were asked to adopt the same freely chosen pedaling cadence (resulting in 67.3 ± 5.7 rpm and corresponding to 120.6 ± 17.1 W of power output) with a comfortable seated position on the saddle. Such a protocol was chosen in order to avoid the occurrence of any sign of neuro-muscular alterations due to fatigue (Conforto and D'Alessio, 1999), which could negatively bias the execution of the exercises (Castronovo et al., 2012).

### sEMG recordings

sEMG data were recorded from the following eight muscles of the dominant leg, defined as the leg the subjects usually used to kick a ball: Gluteus Maximus (Gmax), Biceps Femoris long head (BF), Gastrocnemius Medialis (GAM), Soleus (SOL), Rectus Femoris (RF), Vastus Medialis (VAM), Vastus Lateralis (VAL), and Tibialis Anterior (TA). These muscles were chosen because they are deemed representative of the main muscular groups acting across the three main degrees of freedom involved in cycling, which are hip flexion-extension (RF, Gmax, BF), knee flexion-extension (RF, VAL, VAM, BF, GAM), and ankle plantar-flexion (GAM and SOL) and dorsi-flexion (TA) (So et al., 2005; Hug and Dorel, 2009). A pair of Ag/AgCl electrodes was applied on each muscle, following the SENIAM recommendations (Hermens et al., 2000). Before applying the electrodes, the skin was shaved and cleaned to improve the electrode/skin impedance. sEMG data were collected with a wireless system (BTS FREEEMG 300, BTS s.p.a.) equipped with eight bipolar wireless channels, sampled at 1000 samples/s and digitized at 14 bits. All the sEMG signals were synchronized with the force data coming from the instrumented pedals. Preliminary results including part of these data were previously published (De Marchis et al., 2012).

### Data preprocessing

sEMG signals were filtered in the band (20–450) Hz, preprocessed for noise removal (Conforto et al., 1999), full-wave rectified and low-pass filtered at 4 Hz with a 3rd order Butterworth filter to obtain the signal amplitude envelope (Neptune et al., 2009). Each muscle pattern was amplitude normalized to the maximum value of the envelope across the two pedaling conditions (i.e., PPT and EPT). Time scale was normalized by interpolating each sEMG envelope and each force component for each cycle on 100 data points, each one representative of the integer percentage of the pedaling cycle. A pedaling cycle was defined as the complete revolution of the crank starting from Top Dead Center (TDC, 0°), passing through Bottom Dead Center (BDC, 180°) and back to TDC in a 360° cycle.

### Muscle synergies extraction

Muscle synergies were extracted by means of Non-negative Matrix Factorization (NMF) (Lee and Seung, 1999) applied to the matrix ***M*** containing the envelopes of the eight muscles: the algorithm looks for an approximate solution of the kind *M* ≅ *WxH* by minimizing the matrix norm ||*M−WH*||, where ***M*** is the initial matrix containing the envelopes of the signal from each muscle, ***W*** is a 8×***s*** matrix of the synergy vectors and ***H*** is a matrix containing the time-varying activation profiles, ***s*** being the number of modules specified before the NMF application. The convergence is ensured by the use of multiplicative update rules for each iteration of the algorithm.

The applied procedure followed the hypothesis that the number of synergies is not fixed from cycle to cycle, but the subjects can select a subset of modules belonging to the space of the possible basis vectors. This is particularly true for the feedback condition, in which the subjects adopt a new pedaling technique and may thus explore the space of muscle coordination to accomplish the biomechanical demands. We did not make any assumption about the similarity between modules, so that the dimensionality of the space of the basis vectors explored by the subjects is a-priori unknown.

For each subject and for each pedaling condition (PPT and EPT), the entire data set was divided into multiple episodes with each containing three consecutive cycles. A set of muscle synergies was then extracted from every of these episodes. The number of muscle synergies ***s*** for the reconstruction of the matrix ***M*** for each episode was chosen by calculating the Variance Accounted For (*VAF*) by the reconstruction *WxH* for each muscle activation profile, defined by Equation 6:

(6) $$VAF_{i}=\frac{\sum_{j=1}^{k}{\left(M_{ij}-R_{ij}\right)}^{2}}{\sum_{j=1}^{k}{\left(M_{ij}\right)}^{2}}$$

where ***R*** = *WxH* is the matrix emerging from the synergy model, ***k*** is the number of samples and ***i*** indicates the muscle taken into account for *VAF_*i*_* calculation. The number of extracted synergies ***s*** was varied between 1 and 8 for *VAF_*i*_* calculation, and ***s*** was chosen as the smallest number able to explain at least the 90% of the variance for each muscle. This approach is stringent enough to ensure a proper reconstruction of the original EMG signals in each cycle.

All the extracted modules were then pooled across subjects and conditions in order to obtain the whole synergy matrix ***W***_**all**_ containing all the extracted synergy vectors from all the pedaling cycles for all the subjects.

### Synergy discovery

A synergy discovery procedure was then applied to the matrix ***W***_**all**_ by performing a 2-dimensional non-linear Sammon's mapping (Sammon, 1969): briefly, this analytic procedure is based on mapping a dataset of k L-dimensional vectors belonging to the L-space (i.e., the 8-dimensional space of muscle synergy vectors in this study) to set of k N-dimensional vectors in the N-space (with *N* < *L* and usually set to *N* = 2 or 3) by preserving the inherent data structure. The inter-point distance defined in the L-dimensional space is maintained in the projected N-dimensional space by using an error minimization procedure. This is achieved by minimizing an error criterion which penalizes differences in distances between points in the original L-space and the corresponding points in the projected N-space. The error function to be minimized is defined as follows:

(7) $$E=\frac{1}{\sum_{i=1}^{k-1}\sum_{j=i+1}^{k}d_{ij}}\sum_{i=1}^{k-1}\sum_{j=i+1}^{k}\frac{{\left(d_{ij}-d_{ij}^{*}\right)}^{2}}{d_{ij}}$$

where *k* is the number of vectors in both the original and projected dataset, *d*_*ij*_ is the Euclidean distance between the *i*-th and *j*-th points in the L-space, d^*^_ij_ is the Euclidean distance between the *i*-th and *j*-th points in the N-space. The error function is minimized using a second order steepest descent procedure (Sammon, 1969; De Ridder and Duin, 1997).

This procedure has been followed with the aim of establishing the dimensionality underlying ***W***_**all**_, that is the number of representative modules. The interpretation and clustering of the Sammon's 2-D distribution allows a quantification of the number of underlying basis vectors, by observing their groupings on the 2-D map.

The number of underlying basis vectors composing ***W***_**all**_, was obtained by applying a hierarchical clustering (Ward minimum variance method, Matlab Statistics Toolbox) to the Sammon's map values, in order to organize it in clusters in the 2-D space. These clusters were used to group the synergy vectors contained in ***W***_**all**_, and the representative basis vectors were calculated as the average ***W*** within each cluster, leading to the representative basis vectors ***W***_**basis**_.

### Synergy activation analysis

After applying the clustering, we performed a Non-negative Reconstruction (NNR, Muceli et al., 2010) on all the consecutive cycles (60 in average for each trial) for each subject and for each pedaling condition: NNR consists of applying NMF by keeping ***W*** fixed and letting ***H*** update at each algorithm iteration with the following rule:

(8) $$H_{rc}\leftarrow H_{rc}\frac{{\left(W_{\text{basis}}^{T}M\right)}_{rc}}{{\left(W_{\text{basis}}^{T}W_{\text{basis}}H\right)}_{rc}}$$

where the indexes *r* and *c* are referred to each component of the defined matrixes, and *T* denotes the transposed matrix. The temporal activation ***H*** for each component of ***W***_**basis**_ provides information about the recruitment of that synergy within the trial cycle by cycle. The information related to the recruitment ***H*** of each component of ***W***_**basis**_ was put in relation to the index *IE* cycle by cycle. The amount of activation of a synergy was expressed as the area underlying the temporal profile of activation within each cycle. The ability of the ***W***_**basis**_ to reconstruct the repertoire of muscle activations from the set of all the consecutive cycles for all the subjects was evaluated by determining *VAF*_*i*_ for each muscle.

### Statistical analysis

Differences between force profiles, indexes of effectiveness, muscle activation patterns, and muscle synergy activation coefficients were assessed by using a one-way ANOVA with conditions (PPT and EPT conditions) as factors, with statistical significance coming from *p*-values lower than 0.05. All the differences were evaluated in four different sectors of the pedaling cycle, with each sector defined according to Hug et al. (2008), and roughly corresponding to the following: 1st sector is around TDC, 2nd sector refers to the down-stroke phase, 3rd sector roughly corresponds to the part around BDC, 4th sector is related to the up-stroke phase, with the corresponding EMG sectors defined by taking into account the electromechanical delay (Conforto et al., 2006; Hug et al., 2008).

In order to check that each obtained NNR reconstruction is significantly different from that expected from chance, we used a procedure similar to that used in Cheung et al. (2012): we compared the obtained *VAF*_*i*_ reconstruction values with those expected from chance ***VAF***_**shuffle**_: for each reconstruction, 100 random synergy vectors were generated by shuffling the muscle components of each ***W***_**basis**_ vector, and the obtained ***VAF***_**shuffle**_ values expected from chance were compared with the reference *VAF*_*i*_ reconstruction value.

## Results

In this section, pedal force profiles are shown for the two pedaling conditions, and the corresponding possible changes in the EMG profiles and the underlying muscle synergies are reported as potential signs of neuromuscular adaptations to an altered pedaling technique.

### Modification of the force profiles

**Figure 3:**
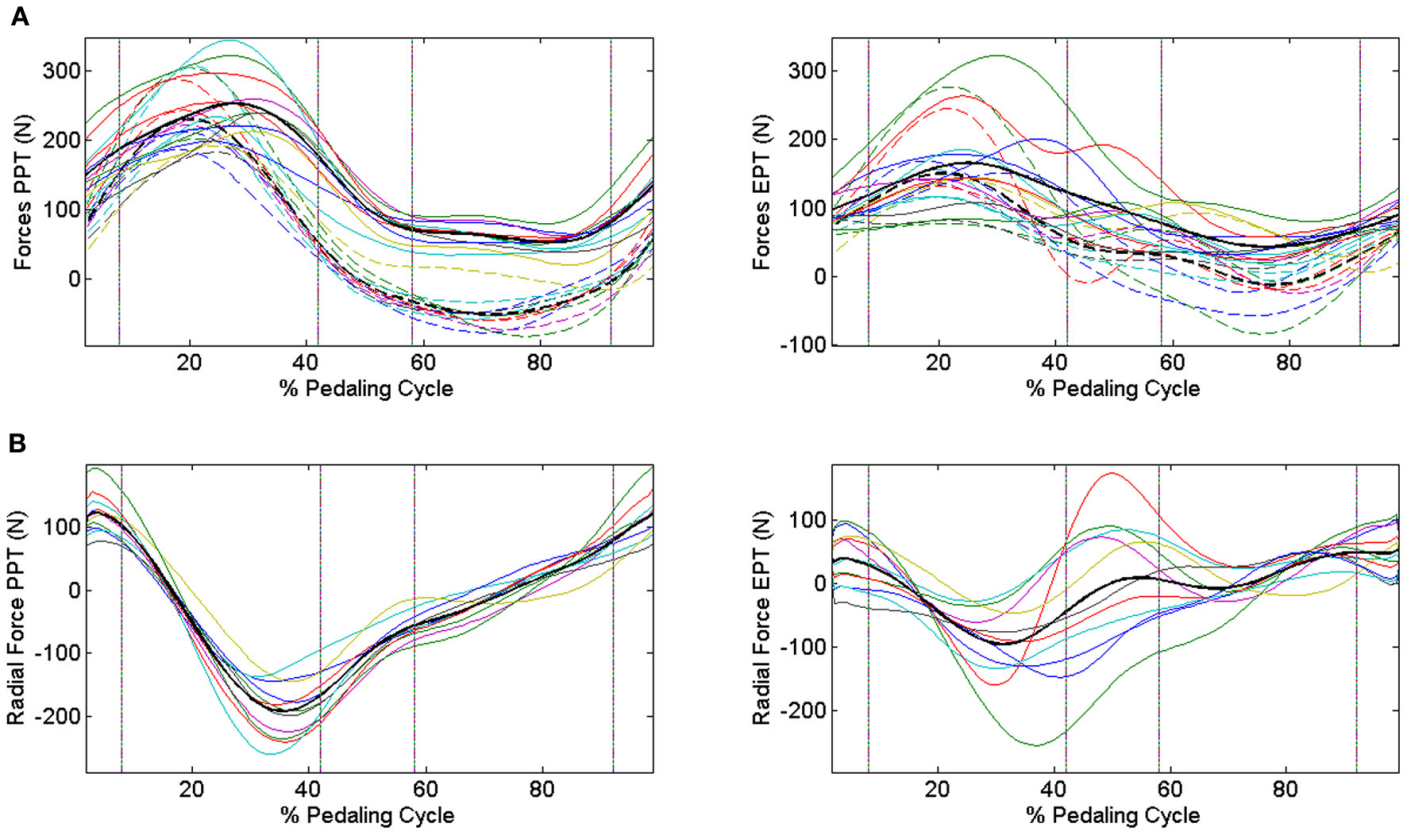
**Modifications in the force profiles when changing the pedaling technique through *IE* feedback.** Upper panels **(A)** Dashed lines represent tangential components, continuous lines represent total force components, and bold black lines are the profiles averaged across subjects. Different colors refer to different subjects. PPT condition left panel: *F*_tg_ follows the trend of *F*_tot_ only in the down-stroke phase. EPT condition right panel: subjects improve their mechanical effectiveness, projecting the forces in such a way to lead *F*_tg_ to approach *F*_tot_ also during the pull-back and pull-up phases. Lower panels **(B)** Radial force components in the two conditions. PPT condition left panel: distribution of the dissipated forces is spread over a wide range of values. EPT condition right panel: radial components are reduced within a narrower range around 0 N, highlighting the improvement in the pedaling strategy. Black lines represent average *F*_rad_ profiles.

**Figure 4:**
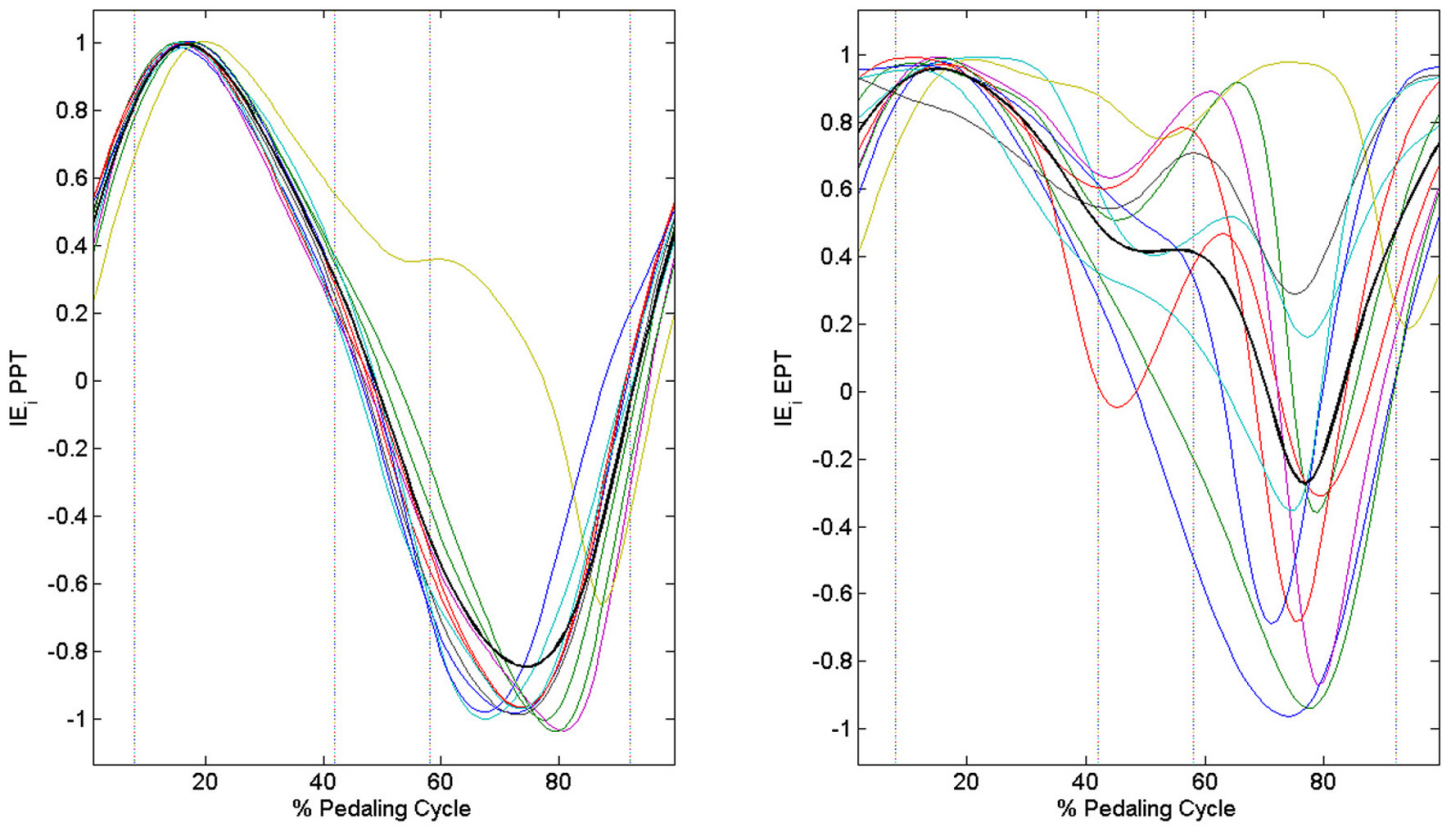
**Instantaneous index of effectiveness across the integer percentages of the cycle.** PPT **left panel:** *IE*_*i*_ becomes negative in the second part of the pedaling cycle. EPT **right panel:** *IE*_*i*_ values increase in the second part of the pedaling cycle.

When passing from the PPT condition to the EPT one there is a significant improvement in *IE* (*IE*_PPT_ = 0.41 ± 0.09, *IE*_EPT_ = 0.68 ± 0.14, *p* < 0.005). As outlined in Figure 3A, feedback of mechanical effectiveness helps the subjects to effectively orient the forces on the pedal, leading the profile of *F*_tg_ to follow the trend of *F*_tot_ along all the cycle, while this happens only in the down-stroke phase during the PPT condition. The effective force application mainly consists of a reduction of the average *F*_tot_ value along the first half of the pedaling cycle and an increase in *F*_tg_ during the second half (*p* < 0.005); this action is associated with the reduction in amplitude of the radial force component profile *F*_rd_ along all the cycle, as shown in Figure 3B. This action is reflected on the instantaneous index of effectiveness (*IE*_*i*_), showing an increased level in the second part of the cycle, in particular for sectors 3 and 4 (Figure 4).

### Neuromuscular adaptations

**Figure 5:**
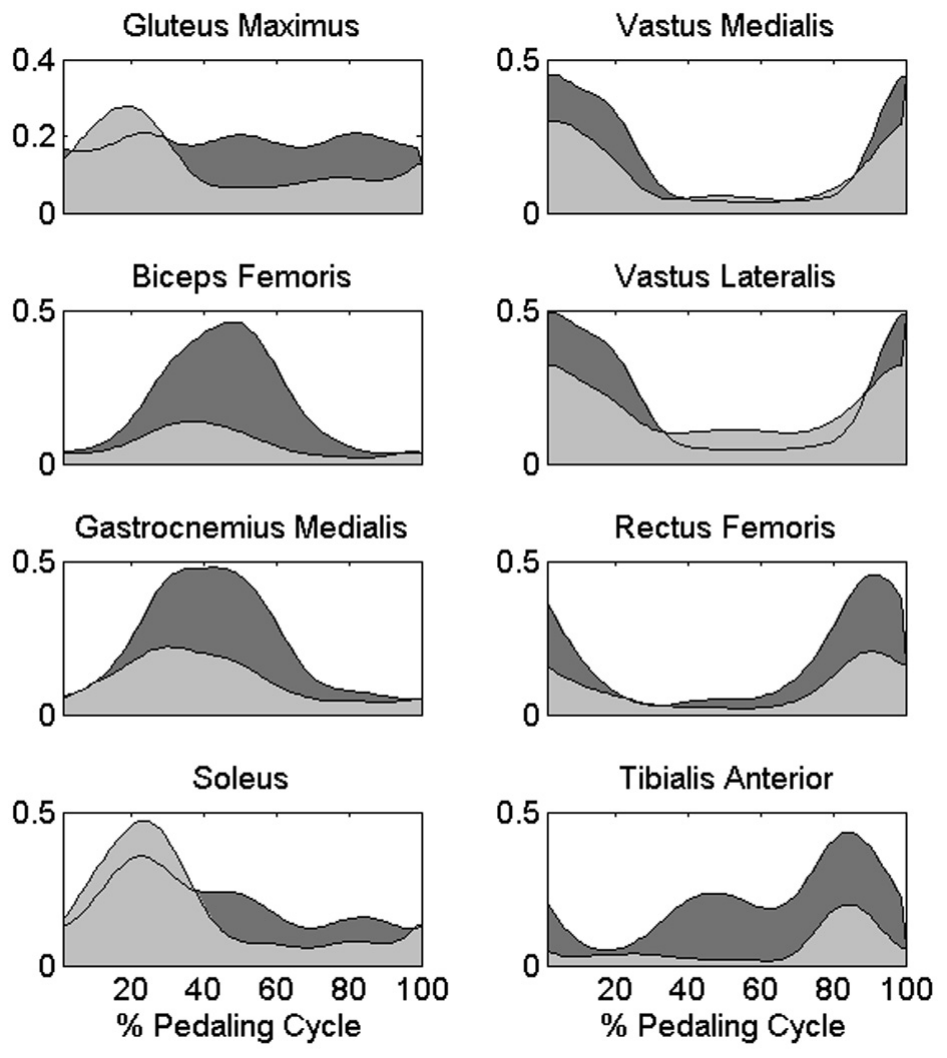
**Differences in the muscle activation profiles between PPT and EPT pedaling conditions.** Group average muscle activation profiles are reported as filled areas: (PPT: light gray area, EPT: dark gray area). y-axis represents the activation level with respect to the reference normalization value of each muscle in arbitrary units.

The change in the distribution of the force profiles described in the previous section is accompanied by some adaptations in the activation of each muscle, particularly evident as an increased level of activity for BF, GAM, RF, and TA (Figure 5), a reduction in the activity of the mono-articular knee extensors (VAM and VAL) in the 1st sector, and an increased tonic activity for Gmax and SOL muscles. (Statistically significant values reported in Table 1).

**Table 1:**
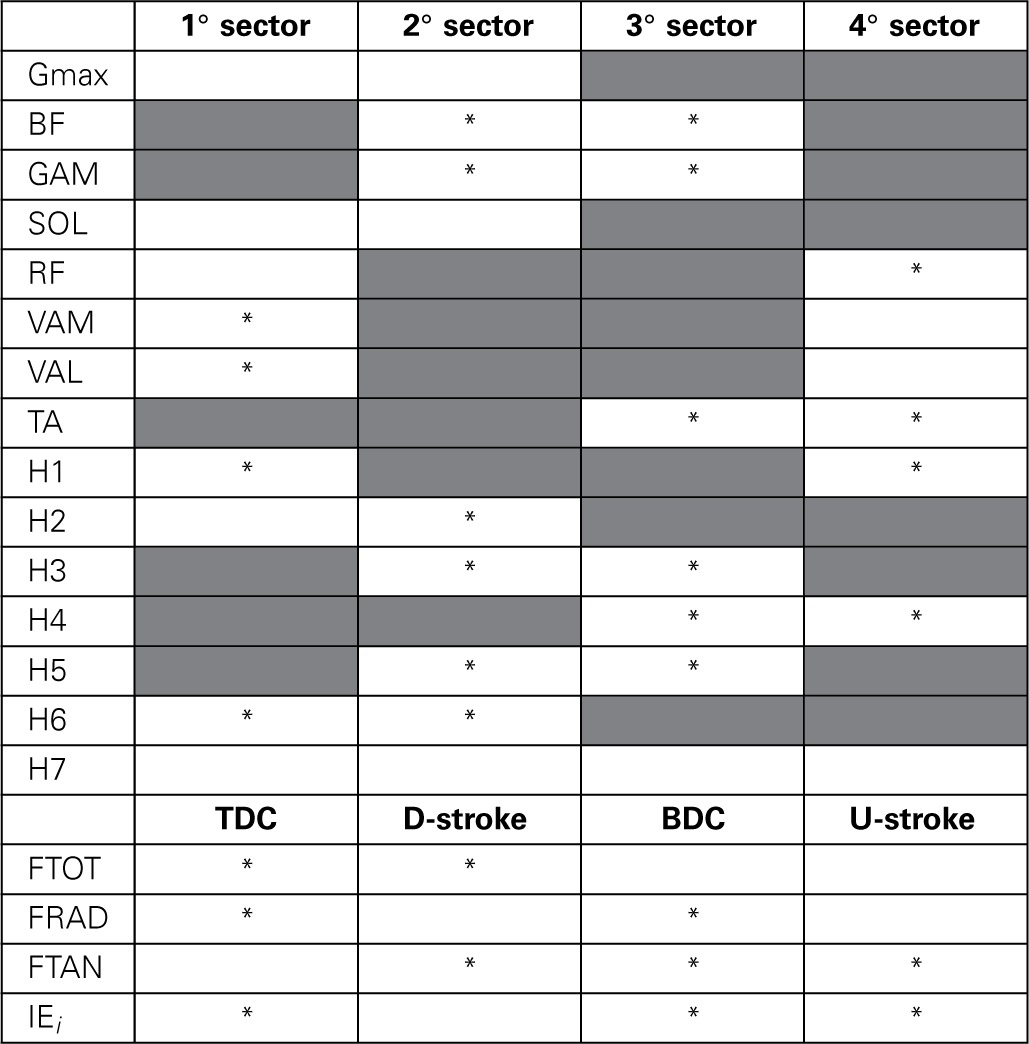
**Neuromuscular adaptations passing from PPT to EPT by taking into account the average amount of muscle activity as the area underlying the profile of the muscle activations and the synergy activation coefficients**.

Force profiles modifications refer to the change in the mean value in each sector. A statistically significant difference in the amount of activation and in the force profile (p < 0.05) is indicated by ^*^. Dark gray rectangles indicate that muscle/synergy is not active in that particular sector.

**Figure 6:**
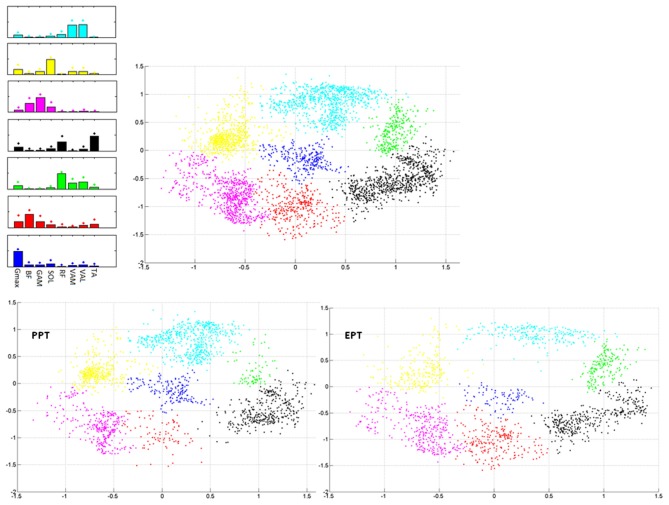
**Sammon's maps. Right upper panel**: Sammon projections of *W*_all_. Different colors refer to the different identified clusters on the map. Each point corresponds to the projection of a single synergy vector of *W*_all_. **Left upper panel**: 8-D average synergy vectors (mean + SD in figure) among the elements of *W*_all_ belonging to the 2-D clusters identified on the map. **Lower panels:** Sammon's distribution related to the synergy vectors of *W*_all_ extracted from the PPT (left panel) and EPT (right panel) conditions.

During the cycle by cycle synergies extraction, from 3 to 5 synergies were extracted, and they were used to populate the matrix ***W***_**all**_. Seven clusters were indentified from the 2-dimensional Sammon's projections of ***W***_**all**_ pooled across the two pedaling conditions (Figure 6). The corresponding centroids ***W***_**basis**_ in the 8-dimensional space contain information about the structured information in the data. When passing from PPT to EPT there is a stability in the location of the clusters on the map; two zones (red and green in Figure 6) were more populated in EPT, meaning that additional basis vectors are explored in the EPT condition.

**Figure 7:**
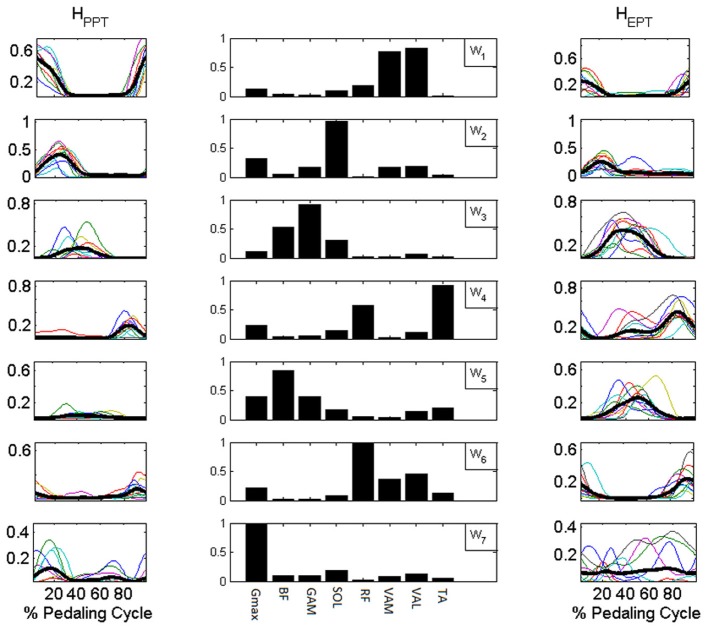
**Synergy activation coefficients H extracted with NNR by using *W*_basis_. Central column**: components of *W*_basis_. **Side columns**: synergy activation coefficients extracted for the reconstruction of the muscle activation patterns of the PPT condition (*H*_PPT_ left column) and of the EPT condition (H_EPT_ right column). Subjects switch between additional modules to accomplish the different mechanical requirements of the EPT pedaling condition.

Synergy activation coefficients *H* (Figure 7) were obtained by applying NNR on each set of consecutive cycles (60 in average for each subject) by keeping ***W***_**basis**_ fixed. The NNR allowed a reconstruction with mean *VAF_*i*_* values for each muscle always higher than 0.9 (except for Gmax, presenting anyway a satisfying reconstruction level). All the obtained *VAF* values were significantly higher than those expected from chance by applying NNR with ***W***_**shuffle**_ (*p* < 0.01 for each muscle in Table 2).

**Table 2:** ***VAF*_*i*_ values and *VAF*_shuffle_ values**.

	***VAF***_***i***_	***VAF*_**shuffle**_**
Gmax	0.88 ± 0.13	0.72 ± 0.09
BF	0.95 ± 0.04	0.71 ± 0.06
GAM	0.98 ± 0.01	0.76 ± 0.04
SOL	0.98 ± 0.01	0.78 ± 0.02
RF	0.92 ± 0.07	0.70 ± 0.07
VAM	0.96 ± 0.01	0.77 ± 0.02
VAL	0.97 ± 0.01	0.79 ± 0.03
TA	0.97 ± 0.01	0.69 ± 0.14

There is a statistically significant difference between the VAF_*i*_ values obtained by reconstruction with W_basis_ and VAF_i_ values obtained from shuffled versions of the original basis vectors.

In the PPT pedaling condition only the first four modules (***W***_***1–4***_, Figure 7) showed a significant level of activity. The first module ***W***_***1***_ consists of the co-activation of two mono-articular knee extensor muscles (VAM and VAL) and a bi-articular one (RF, also crossing the hip joint), and it is active during the first part of the pedaling cycle. ***W***_***2***_ mainly consists of the activity of two ankle plantar-flexors (SOL and GAM) together with G_max_, and it is active within the first quarter of the cycle. ***W***_***3***_ involves the co-activation of two bi-articular knee flexors (BF and GAM), and it is active around BDC. ***W***_***4***_ is composed by the activity of RF and TA and intervenes in the last quarter of the cycle before TDC.

In the EPT condition subjects showed an altered recruitment of the synergies active in PPT, and use additional synergies belonging to ***W***_**basis**_. ***W***_***1***_ and ***W***_***2***_ are less active (*p* < 0.05, Table 1), while ***W***_***3***_ and ***W***_***4***_ show an increased level of activation within the functional sectors in which they are recruited (*p* < 0.005). The change in the distribution of the values on the Sammon's map consists on the activation of two additional modules. ***W***_***5***_ consists of the co-activation of knee flexor muscles and TA, and it is active within sectors 3 and 4. ***W***_***6***_ occurs just before TDC and it is mainly composed by a merging of synergies ***W***_***1***_ and ***W***_***4***_(RF, vastii and TA). ***W***_***7***_ mainly reflects the tonic activity of Gmax and SOL during the EPT condition.

### Synergy activation coefficients and mechanical effectiveness

The activation of synergies ***W***_***3***_, ***W***_***4***_, ***W***_***5***_ is related to the change in mechanical effectiveness (correlation values in Table 3). The coefficient of variation revealed that these are the only synergies relevantly correlated with *IE*, since they show a lower coefficient of variation *CV* (Table 3), meaning that their behavior is consistent across subjects.

**Table 3:**
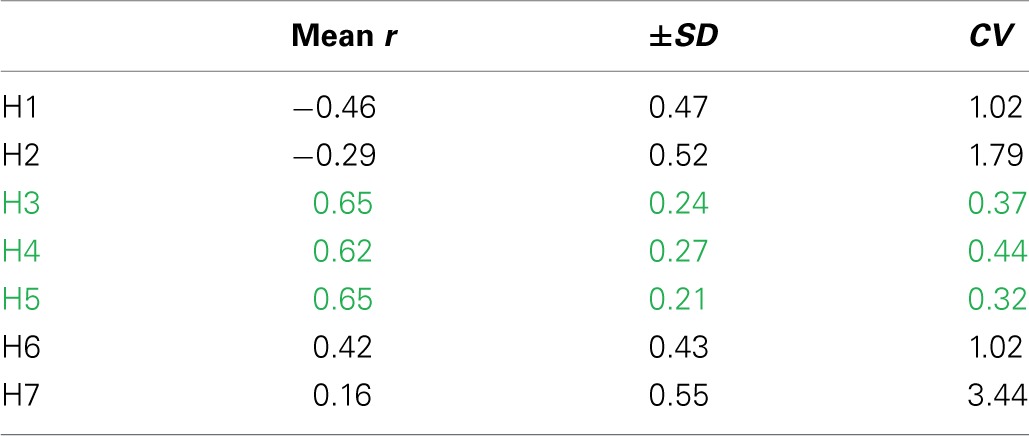
**Correlation between the temporal evolution of the synergy recruitment and the temporal evolution of IE**.

Mean ± SD for each activation coefficient is reported, together with the coefficient of variation CV. H_*3*_, H_*4*_, H_*5*_ are the only components to show a robust behavior across subjects, as indicated by the low CV value.

## Discussion

The obtained results seem to support the existence of a modular motor control in humans, with few muscle synergies shared among different subjects and able to reconstruct the variable muscle activation repertoire shown under different pedaling conditions. Pedal force measurements together with the use of a visual feedback of mechanical effectiveness allowed a controlled change in the pedaling strategy, which resulted in the ability of orienting the pedal forces in a direction almost completely tangential to the circle spanned by the pedal, thus confirming the validity of the used protocol.

When the subjects chose their PPT, they adopted a strategy which was mainly based on the propulsive action during the down-stroke phase (TDC – BDC, 0–180°), where the tangential component of force is almost coincident with the total force, while *F*_tg_ becomes negative during the second part, meaning that the action of the leg slightly opposed the propulsive action, so that most of the propulsion was generated by the down-stroke action of the other leg. Besides *F*_tg_, a dissipated radial component of force *F*_rd_ was present all over the cycle. The obtained values of *IE* are in line with previous studies measuring mechanical effectiveness during pedaling with a self-selected strategy (Sanderson, 1991; Mornieux et al., 2006; Zameziati et al., 2006).

When the subjects adopted an effective strategy, *F*_tg_ tended to follow the profile of *F*_tot_ also in the second part of the cycle (BDC – TDC, 180–360°), and this action was accompanied by a reduction in *F*_tot_. This behavior can be associated with the reduction of the radial force component resulting in a significant increase of the index of mechanical effectiveness *IE*.

### Adaptation in the modular control of pedaling across different pedaling techniques

A episode-by-episode synergy extraction procedure and the subsequent clustering on the Sammon's non-linear projection allowed the identification of seven basis muscle synergy vectors. In order to satisfy the mechanical requirement the subjects switched between the available modules to form different motor programs (Kargo and Nitz, 2003).

Pedaling with a low mechanical effectiveness was accomplished by using a modular muscle coordination mainly characterized by four muscle synergies which were able to account for most of the variance of the EMG data.

Passing to a mechanically EPT resulted in a modification of the mechanical demand which was accompanied by a modification in the muscle activation patterns with respect to the PPT condition, and additional modules were activated to explain the variance of the data. We therefore speculate that these additional muscle synergies may represent a neural mechanism reflecting short term adaptation, where the subjects tend to adopt the already learnt muscle coordination shown in PPT, and they add modules to achieve the imposed mechanical requirement.

### Functional interpretation of the muscle synergies

The structure of the extracted muscle synergies may be associated to different biomechanical sub-functions during the pedaling cycle (Figure 7):

***W***_***1***_, mainly consisting of knee extensors activity (VAM, VAL, RF), acts during the first part of the cycle and it is key to power production during the down-stroke phase, when the knee joint passes from a flexed position (TDC) to an almost completely extended one (BDC).

***W***_***2***_ involves the co-activation of SOL, GAM, and G_max_. The main action of the two ankle plantar-flexors (SOL and GAM) may be responsible for the ankle angle variations during the pedaling cycle. This synergy might thus contribute to the fine control of the ankle movement preparing the pull back phase.

***W***_***3***_ is characterized by the activity of two bi-articular knee flexors (BF and GAM), and it starts just before BDC, when the knee joint begins its flexing action in the second part of the cycle.

***W***_***4***_ is a synergy characterized by the co-activation of RF and TA, and it intervenes during the last quarter of the cycle, in the transition phase between up-stroke and down-stroke passing around TDC, during the hip flexion action, propelling the crank toward the end of flexion.

***W***_***5***_ is a synergy specific for the pedaling condition EPT, mainly consisting of the co-activity of knee flexors (BF and GAM), Gmax and TA muscle and it may be responsible, together with ***W***_***3***_ and ***W***_***4***_, of the pull-up action during up-stroke.

***W***_***6***_ appears in EPT and it seems to consist of a merging between modules ***W***_***1***_ and ***W***_***4***_. It is active during the last part of the cycle and it may be related to an adaptation of the transition phase around TDC. ***W***_***7***_ clearly presents a tonic recruitment along the cycle reflecting the tonic components of Gmax and SOL in EPT.

Passing from PPT to EPT, the activation coefficients ***H***, obtained as a NNR by using ***W***_**basis**_, show some adaptations, involving the amplitudes rather than the timings, which may reflect the satisfaction of the new mechanical requirements imposed by the feedback; this is particularly evident for the synergies *#3* and *#4*, where ***H***_**3**_ and ***H***_**4**_ show a significant increase that contributes to the modifications of the orientation in *F*_tg_, leading to the improvement of the *IE*. ***W***_***5***_ displays a level of activation comparable to the one shown by the other synergies, meaning that its action co-participates to the increase of the pedaling propulsion, in particular to power the pedal during the up-stroke phase. On the contrary, ***W***_***1***_ and ***W***_***2***_ which are active during the first part of the cycle, show a reduced activation which may be due to the contribution of the other leg while pulling-up.

The intervention of ***W***_***5***_ is in accordance with what outlined in previous studies (Mornieux et al., 2010), where an increased activity of BF and TA was reported in elite cyclists pedaling with a feedback of mechanical effectiveness. Interestingly, these two muscles were found to be in synergy as well in a mechanically altered pedaling, when they were found to be co-active after a phase shift of the hamstrings activation during backward pedaling in a phase-reversal of the main biomechanical functions (Ting et al., 1999). This aspect might confirm the evidence that ***W***_***5***_ is an available module for the accomplishment of cycling in different conditions.

### Contribution of the modules to mechanical effectiveness

By analyzing the correlation between the synergy activation coefficients cycle-by-cycle and the *IE*, it emerges that those synergies which show an increased activity during EPT (i.e., ***W***_***3***_, ***W***_***4***_, ***W***_***5***_), also show a significant correlation with *IE*, confirming their contribution to the change in the pedaling technique.

### Evidence of a between-task shared modular organization: the case of human walking

**Figure 8:**
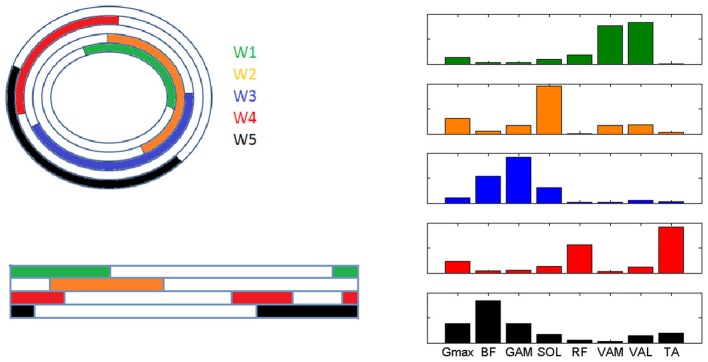
**Graphical representation of the intervals of recruitment of the 5 muscle synergies characterizing the analyzed pedaling conditions (PPT and EPT, *W*_1-5_ represented outwards in color code) and the corresponding recruitment of the synergies in the study by Neptune et al. (2009) (*W*_1-5_ represented downwards with the same color code, step cycle indicated from one heel strike to the next one)**.

The fascinating hypothesis that some muscle synergies can be task-specific and other may be shared between different tasks (d'Avella and Bizzi, 2005), seems interestingly confirmed in our study. In particular, 4 out of the 7 synergies identified in the present study are highly similar to those extracted in other studies of human walking (***W***_***1***_, ***W***_***2***_, ***W***_***4***_, ***W***_***5***_), but they are recruited in a different order during the movement cycle between the two tasks (Figure 8). This similarity is more evident when the comparison is carried out with studies using the same decomposition technique (Neptune et al., 2009; Clark et al., 2010), so that a common interpretation can be drawn. According to the modules extracted in (Neptune et al., 2009; Clark et al., 2010), ***W***_***1***_ (knee-extensors) is active in walking during the early stance phase providing body support, ***W***_***2***_ (ankle plantar-flexors and gluteus) intervenes during late stance and contributes to swing initiation, ***W***_***4***_ (RF and TA) is recruited just before stance and provides dorsi-flexion during and just after heel strike, ***W***_***5***_ (Hamstrings and TA) decelerates the leg at the end of swing.

It is worth noting that two completely different tasks like walking and cycling, which basically involve the same body segments in the lower limbs, share some modules, but these modules are activated in a different way in order to satisfy the current task requirements in terms of both kinematics and kinetics. While ***W***_***3***_(BF and GAM) seems to be specific for a task like cycling, ***W***_***5***_ seems to be shared with walking but it appears in cycling only when a change in the biomechanical requirement is present, providing further evidence that a small set of motor modules can account for a variety of motor tasks through a simple selective activation and combination of modules. This aspect, related to motor adaptation, is in accordance with the theoretical functioning of the structure of a modular controller, since the use of an already existing module allows a faster adaptation to a perturbation in the task that is likely to be compatible with the modules (d'Avella and Pai, 2010).

### Aspects related to training and rehabilitation

With respect to the study carried out on trained cyclists (Hug et al., 2010), where 3 synergies were extracted, here we extracted 4 synergies in the PPT. Despite the possible effect of the EMG processing techniques and the criterion used to choose the number of modules, a possible explanation could be attributed to the different power output expressed by the two studied populations, since an higher power output would increase the signal-to-noise ratio and would lead to a reduced number of synergies explaining an higher amount of *VAF*. An alternative hypothesis is that the difference in the number of synergies may be also due to a possible reorganization in the recruitment of the modules in trained subjects (Chapman et al., 2008), mainly consisting in the simultaneous recruitment of the modules ***W***_***2***_ and ***W***_***3***_, and this may be a sign of the differences between the two studied populations of cyclists. Even if the merging of motor modules has been observed for stroke patients and it was able to explain the main biomechanical impairments during upper and lower limb movements (Clark et al., 2010; Cheung et al., 2012), up to now it is not known if a simultaneous recruitment of separate motor modules is feasible in healthy conditions, and if it can lead to improved performance in terms of metabolic or muscular efficiency as a consequence of expertise.

The possible spatio-temporal re-organization of the modules could be studied for the functional evaluation of the cycling performance in both healthy and pathologic conditions. For example, this could emerge from relating the adaptations in modularity with the evolution of physiological factors such as muscular efficiency (Zameziati et al., 2006) or muscle fatigue (Theurel et al., 2011), or from providing a neuro-rehabilitation program based on cycling (Ambrosini et al., 2012), by relating the changes in modularity to the changes in the mechanical outcome of movement.

### Possible methodological limitations

The methodological approach used in the present study, consisting on the extraction of muscle synergies on an episode-by-episode basis, is subject-specific and is able to highlight intra-subject variation in muscle synergies. Nevertheless, it may not be able to characterize the behavior of the participant sample as a whole, since it might fail in capturing common features that could emerge only by decomposing data pooled across subjects and conditions.

Another possible limitation might rely in the use of the synchronous synergy model: in fact, up to now, it is not known whether the application of the time-varying synergy model could extract features otherwise not accessible when studying cyclic movements of the lower limbs.

## Conclusions

Our results provide further evidence that the motor system might rely on the combination of a reduced number of motor modules for the control of movement. A small number of synchronous muscle synergies, scaled in amplitude and adjusted in time, are able to account for most of the variance of the EMG data. These modules are shared among subjects and across modifications in the mechanical requirements for the execution of the pedaling gesture imposed by the feedback, with the main adaptations occurring in those modules deemed responsible for a particular biomechanical sub-function (i.e., pulling up during the up-stroke phase).

Adapting to a new pedaling technique imposed by the feedback seems to be accomplished by exploring an already learnt modular structure, which is not pedaling-specific, but it is mostly shared with the one generally found in human gait. This aspect opens further perspectives in neuro-rehabilitation, e.g., by inserting cycling-based programs for the functional recovery of pathologic gait.

With respect to the study carried out by Kargo and Nitz (2003), where it was shown that skill learning is achieved by increasing the probability of selecting the most efficacious motor programs, our study only took into account a very reduced time slot of exercise, so that an immediate effect of training on a possible tuning of muscle synergies is not visible. Based on the previous observations, further studies should analyze the effect of short or long periods of training with biofeedback on the structure of muscle synergies in cycling, in order to establish if a modification in modularity occurs by altering the structure of the synergy vectors, or by selecting different motor programs. Aiming at this, and in order to overcome possible limitations of the present study, also the inter-limb coordination should be taken into account.

### Conflict of interest statement

The authors declare that the research was conducted in the absence of any commercial or financial relationships that could be construed as a potential conflict of interest.
